# Evaluating vectors for the design of a spillover-disrupting Lassa virus transmissible vaccine

**DOI:** 10.1371/journal.pcbi.1014390

**Published:** 2026-06-26

**Authors:** Scott L. Nuismer, Christopher H. Remien, Bruno Ghersi, Jenna Nichols, James Bangura, Emmanuel Amara, Marilyn C. Kanu, Osman T. Kanu, Edwin G. Lavalie, Mohamed Turay, Patrick L. K. Swaray, Mohamed A. Vandi, Joseph Hughes, Heinz Feldmann, Kyle Rosenke, Michael A. Jarvis, Andrew J. Davison

**Affiliations:** 1 Department of Biological Sciences, University of Idaho, Moscow, Idaho, United States of America; 2 Department of Mathematics and Statistical Science, University of Idaho, Moscow, Idaho, United States of America; 3 Cummings School of Veterinary Medicine, Tufts University, North Grafton, Massachusetts, United States of America; 4 Genetic Design and Engineering Center (GDEC), Bioengineering Department, Rice University, Houston, Texas, United States of America; 5 University of Makeni, Makeni, Sierra Leone; 6 Ministry of Health and Sanitation, Freetown, Sierra Leone; 7 MRC-University of Glasgow Centre for Virus Research, Glasgow, United Kingdom; 8 Laboratory of Virology, Division of Intramural Research, National Institute of Allergy and Infectious Diseases, National Institutes of Health, Hamilton, Montana, United States of America; 9 School of Biomedical Sciences, University of Plymouth, Plymouth, United Kingdom; 10 The Vaccine Group Ltd., Plymouth, United Kingdom; Washington State University, UNITED STATES OF AMERICA

## Abstract

Lassa fever is a viral zoonotic disease that sickens tens of thousands of people each year when it spills over from its rodent reservoir throughout West Africa. Despite the burden this persistent spillover places on public health, stopping it has proven to be an intractable challenge despite decades of investment and effort. A revolutionary solution is the development of a transmissible vaccine that can spread through the rodent population resulting in immune coverage sufficient to eliminate Lassa virus transmission. Here, we evaluate the feasibility of a transmissible vaccine constructed from native cytomegaloviruses (CMVs) previously isolated from the natural reservoir of Lassa virus, *Mastomys natalensis*. Using a combination of field sampling, large-scale CMV genome characterization, and mathematical models parameterized using Bayesian methods, we quantified transmission rates and interactions among viruses in northern Sierra Leone. The results demonstrate that two of the three previously isolated CMVs co-infect freely and have *R*_0_ values consistent with autonomous elimination of Lassa virus from its rodent reservoir. These results set the stage for the development of a CMV vectored transmissible vaccine that could stop the spillover of Lassa virus throughout West Africa.

## Author summary

Lassa fever is a potentially severe viral disease that infects humans living near the mice that serve as the virus’s natural reservoir throughout West Africa. Despite decades of effort, stopping this viral spillover remains an intractable challenge. A revolutionary solution is a transmissible vaccine — a modified, harmless virus that spreads naturally through the mouse population and renders the animals immune to subsequent infection by Lassa virus. By immunizing the mice, human infection can be reduced or prevented. We investigated if Cytomegaloviruses (CMVs) could serve as a vaccine delivery system by analyzing viral genomes collected from wild mice. Fitting the viral genomic data to mathematical models allowed us to quantify how effectively a vaccine constructed from these wild CMV viruses would spread through the mouse population. Of the three CMV viruses we studied, two were predicted to spread sufficiently within the mouse population to eliminate Lassa virus. Our results provide a proof of concept that a transmissible vaccine constructed from these wild viruses is biologically feasible, yielding a scalable solution to the challenge of Lassa fever in West Africa.

## Introduction

Lassa virus infects and sickens tens of thousands of people each year across West Africa [1,2]. Although human-to-human transmission can occur, it is generally rare and restricted to nosocomial settings. Instead, the majority of Lassa fever cases are caused by spillover from the natural host of the virus, the Natal multimammate mouse, *Mastomys natalensis* [3,4]. Human infection is thought to occur through contact with the feces or urine of infected mice, with human cases peaking during the dry season when mice move into human dwellings in search of food [5]. Despite the widespread impact of Lassa virus on human health and its potential to expand its range beyond West Africa, few tools are available for controlling infection and no vaccines are licensed for use in humans [2].

Because humans are infected almost exclusively by contact with infected mice, an alternative to vaccinating humans is to vaccinate the mice themselves. This could reduce the prevalence of Lassa virus within the mouse population and thus limit spillover into the human population. Unfortunately, because of the short lifespan and rapid population turnover of mice, distributing vaccine-laden baits is unlikely to achieve the success of similar programs targeting rabies virus in European foxes and North American raccoons [6]. One possible solution to this problem is to develop a transmissible vaccine that passes from mouse to mouse infectiously, thus avoiding the need to vaccinate each individual directly [7]. Given that the safest approach to a transmissible vaccine is a vector virus expressing a Lassa virus antigen [8,9], a critical first step is the identification of a suitable virus that circulates naturally within the reservoir animal population. Cytomegaloviruses (CMVs), which are large double-stranded DNA viruses in subfamily *Betaherpesvirinae* in the family *Orthoherpesviridae*, are promising in this regard because they are highly species-specific, achieve high prevalence, and appear capable of superinfection [8–10]. Superinfection is a desirable property for a vaccine vector because it minimizes the potential for interference between endemic vector virus and vaccine.

Previous work has characterized the genomes of three different CMVs isolated from *Mastomys natalensis* populations in Mali: *Mastomys natalensis* CMVs 1, 2, and 3 (MnatCMV1, MnatCMV2, and MnatCMV3) [11]. However, it is not known how well these viruses transmit within wild mouse populations or if they interact in ways that could compromise the spread of a vaccine vector. Here we studied the epidemiology of these viruses within a population of wild mice located in the area of Bafodia, a small town in northern Sierra Leone where Lassa virus is endemic. Using extensive mouse sampling, large-scale MnatCMV genome characterization, and mathematical models parameterized using Bayesian methods, we estimated and compared MnatCMV transmission rates and tested for interactions among them. This approach is complementary to previous methods that relied on sequencing prior to virus isolation [12,13], but offers a more granular and refined assessment of vector performance, albeit at a much more restricted spatial scale. We applied this methodology to assess the suitability of the three MnatCMVs for use as transmissible vaccine vectors within reservoir populations of *M. natalensis* in northern Sierra Leone, an area where Lassa fever is endemic and imposes a significant impact on public health.

## Results

### MnatCMV2 and MnatCMV3 circulate in Bafodia

DNA sequence data were generated from oral swab samples taken from captured rodents in the Bafodia area that were identified by DNA barcoding as *M. natalensis*. The data were aligned to a published *M. natalensis* mitochondrial genome and to three large, non-overlapping regions of the published MnatCMV1, MnatCMV2, and MnatCMV3 genomes. A total of 241 animals were confirmed as *M. natalensis* by analysis of mitochondrial DNA sequence data. Applying a calibrated formula linking body length to age yielded an average age of 143.5 days, consistent with prior estimates from studies of *M. natalensis* in Guinea where the average age was 150.2 days [5,14,15]. The MnatCMV data yielded no evidence of infection by MnatCMV1, whereas infection by MnatCMV2 and MnatCMV3 was common, with prevalences of 72.4 (CI: 66.0-78.0) and 49.1% (CI: 44.2-57.5), respectively. Co-infection with both CMVs was also common and observed in 48.7% of mice. The probability of infection by each of these two viruses increased with mouse age (GLM with binomial link; odds 1.01 and $p=7.04\times 10^{-6}$ for MnatCMV2 and odds 1.01 and $p=1.96\times 10^{-7}$ for MnatCMV3), as did the probability of co-infection by both viruses (GLM with binomial link; odds 1.01 and $p=3.10\times 10^{-7}$). Neither sex nor season had a significant impact on the probability of infection by either virus (GLM with binomial link. MnatCMV2 male sex effect: Odds 1.13 and *p* = 0.679. MnatCMV3 male sex effect: Odds 1.64 and *p* = 0.066. MnatCMV2 wet season effect: Odds 1.26 and *p* = 0.474. MnatCMV3 wet season effect: odds 1.43 and *p* = 0.236.).

### *R*_0_ estimates are modest but sufficient

**Fig 1:**
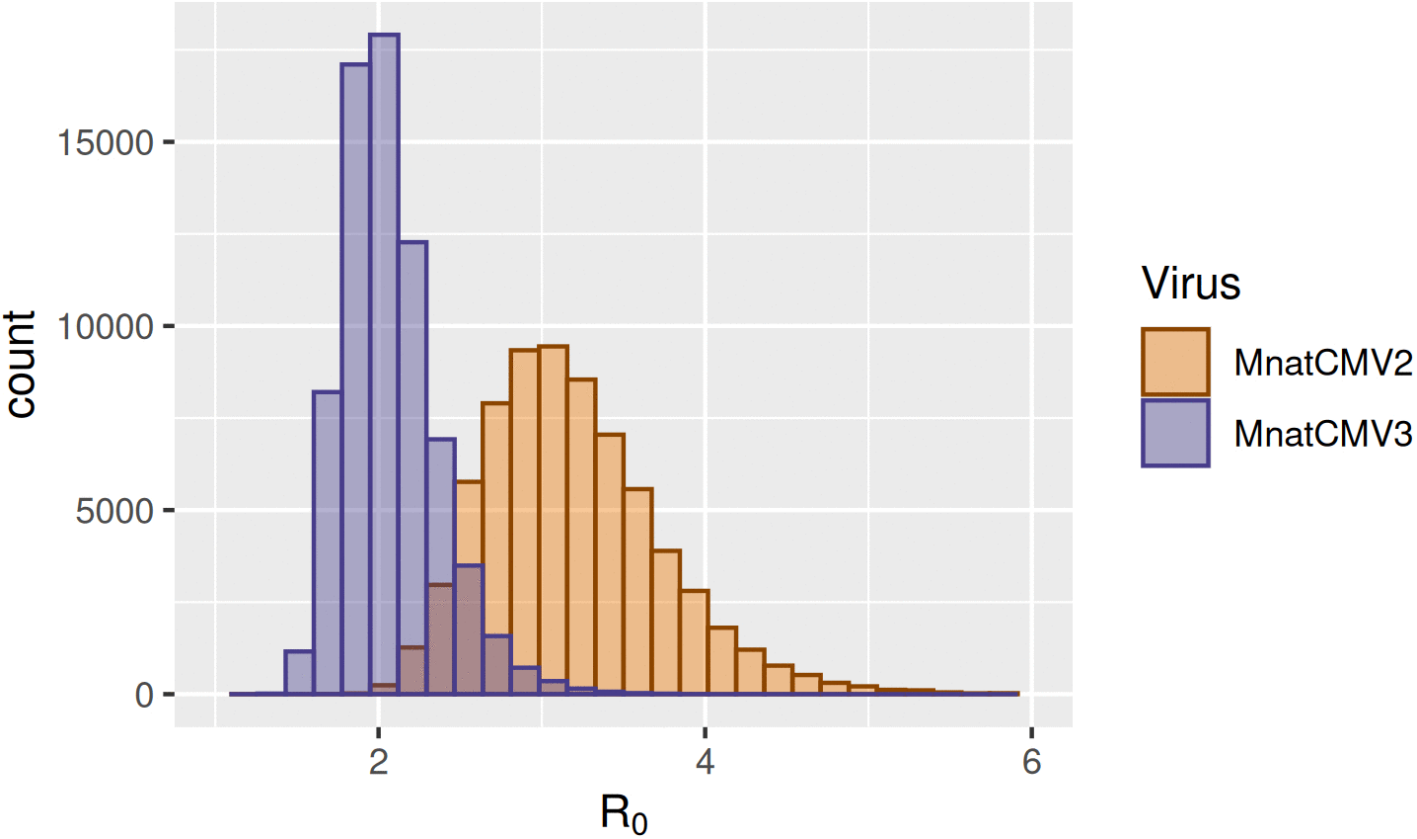
Posterior distributions for the composite parameter *R*_0_ estimated for MnatCMV2 and MnatCMV3.

To investigate the utility of the two MnatCMVs circulating within the *M. natalensis* population of Bafodia as vaccine vectors, we quantified their transmissibility using the composite parameter *R*_0_. We focused on *R*_0_ because previous work demonstrated that pathogen elimination is possible for cases where vaccine *R*_0_ exceeds pathogen *R*_0_ [16]. Analysis of the age-structured infection data using model-based Bayesian inference yielded *R*_0_ estimates equal to 2.99 and 1.96 for MnatCMV2 and MnatCMV3, respectively, with corresponding 95% credible intervals of 2.22-4.27 and 1.58-2.65 (Fig 1). In an idealized scenario, these values are sufficient to eliminate Lassa virus from the reservoir population based on previous estimates showing that the *R*_0_ of Lassa virus within *M. natalensis* populations in nearby Faranah, Guinea ranges between 1.54 and 1.74 [6].

### Superinfection is common

**Fig 2:**
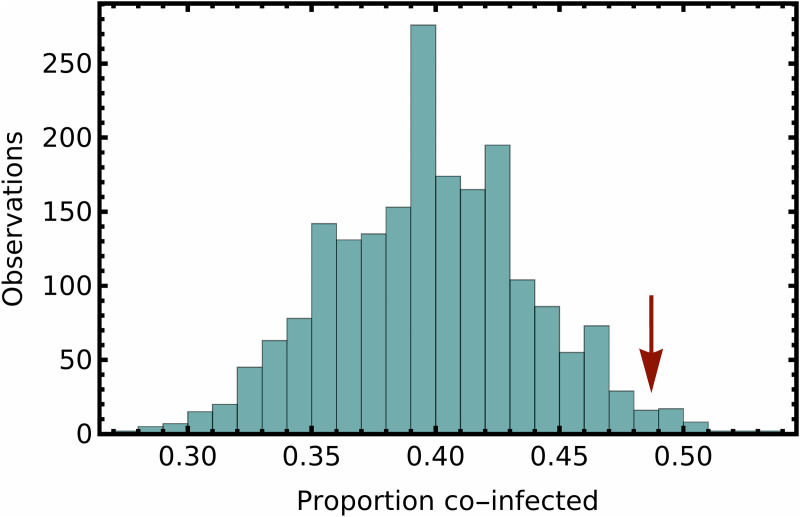
Average levels of co-infection observed in 2000 simulated data sets (histogram) compared to true value of co-infection in the data (marked by the red arrow). 98.3% of simulated values fall to the left of the red arrow.

Previous work has demonstrated that interference among viral vectors, whether driven by immunity or direct competition for host cells, can significantly reduce the efficacy of transmissible vaccines [17,18]. We tested for the presence of interactions between MnatCMV2 and MnatCMV3 by comparing the observed levels of co-infection to those computed from simulated datasets in which interactions between strains were absent. Observed values of co-infection were greater than 98.3% of simulated values (Fig 2), convincingly demonstrating an absence of interference between the viruses. Rather, these results suggest that the two viruses may facilitate one another, with infection by one promoting infection by the other. Alternatively, because we detected and modeled only virus shed into saliva, the high levels of observed co-infection may be attributable to independent factors (e.g., stress) that cause both viruses to emerge from latency and begin shedding simultaneously. High levels of co-infection could also result from shared pathways of transmission among viruses that are attributable to differences in animal behavior or susceptibility to infection. These hypotheses cannot be disentangled using the available data.

### Latency is rare and transient

**Fig 3:**
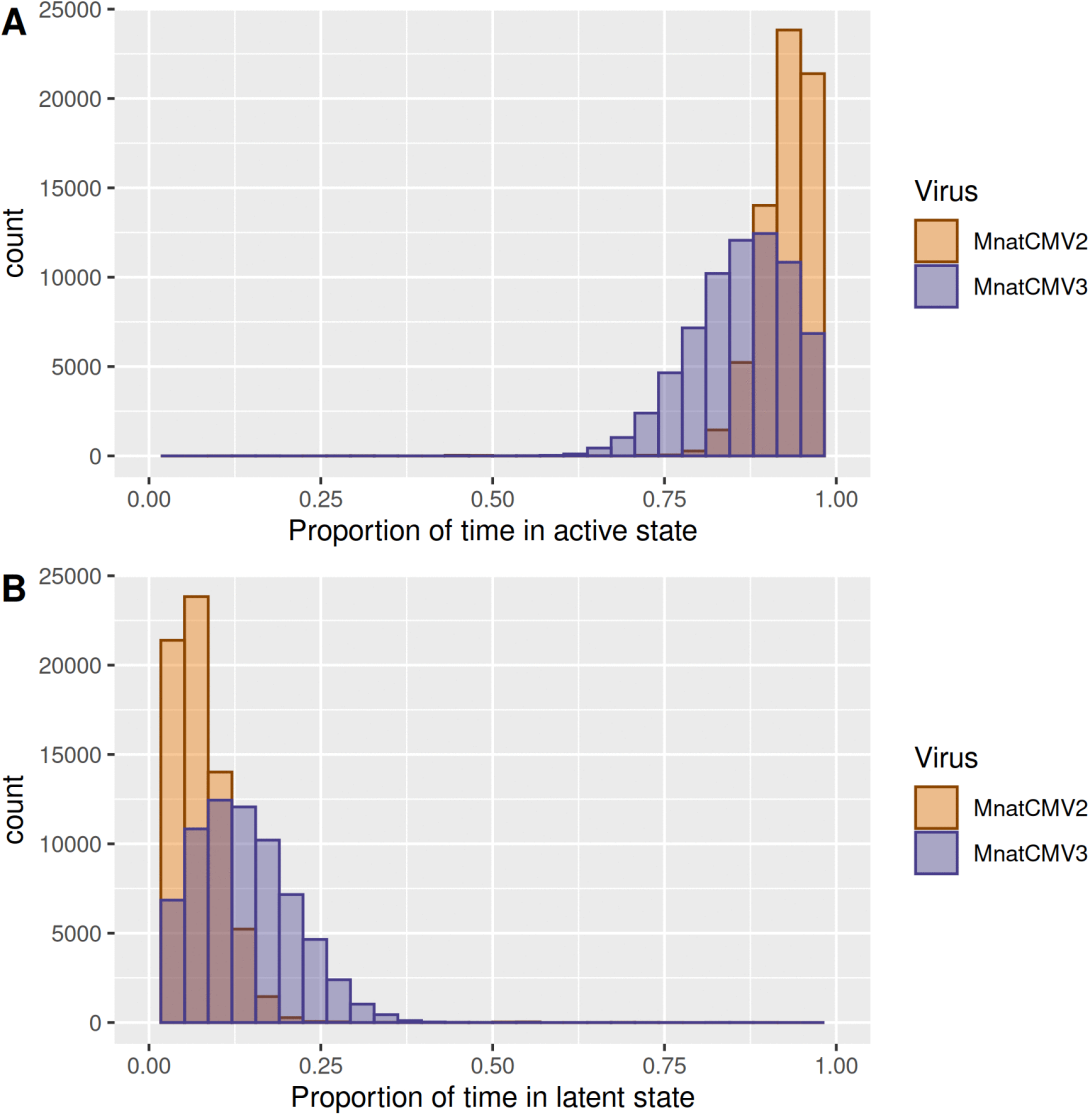
Posterior distributions for the proportion of time animals infected by MnatCMV2 or MnatCMV3 spend in the actively infectious and shedding state (A) and the latent and non-shedding state (B).

CMVs are thought to fall along a continuum ranging from persistent replication and shedding to periodic replication and shedding interrupted by latency [10]. Because latency may influence the rate of spread through the reservoir population and confound efforts to accurately estimate *R*_0_, we integrated transitions between active and latent states into our model and calculations for *R*_0_, allowing us to explicitly estimate transition rates. The results suggest that latency, defined here as failure to shed detectable levels of MnatCMV DNA in saliva, is rare and transient. Specifically, the posterior distribution for the parameter ρ, which measures the rate at which latent infections reactivate and begin to shed virus at detectable levels, yields estimated modal values for MnatCMV2 and MnatCMV3 of 0.059 and $0.070days^{-1}$, respectively. Calculating the expected duration of latency from the full posterior distribution for ρ suggests that latency, when it occurs, lasts, on average, for only 36.4 and 19.4 days, respectively. In contrast, the posterior distribution for the parameter ω, which quantifies the rate of transition to the latent state, yields estimated modal values of 0.002 and $0.004days^{-1}$ for MnatCMV2 and MnatCMV3, respectively. Calculating the expected duration of active infection from the full posterior distribution for ω suggests that active infection lasts, on average, for 389.8 days in MnatCMV2 but only 323.9 days in MnatCMV3. Recognizing that the expected time an infection spends in the actively shedding state is equal to $\frac{\rho }{\rho +\omega }$ allows the posterior distributions for ρ and ω to be used to predict the proportion of time an animal spends in the actively infectious and shedding state. For MnatCMV2, this calculation implies that infected animals spend 92.9% of their time in the actively shedding state whereas animals infected by MnatCMV3 spend 86.4% of their time in this state (Fig 3). Collectively, these results suggest that individuals infected with MnatCMVs experience chronic lifelong infection associated with persistent viral shedding interrupted by only occasional and brief bouts of latency or levels of shedding that fall below the limit of detection.

### MnatCMV2 spreads faster and more broadly than MnatCMV3

**Fig 4:**
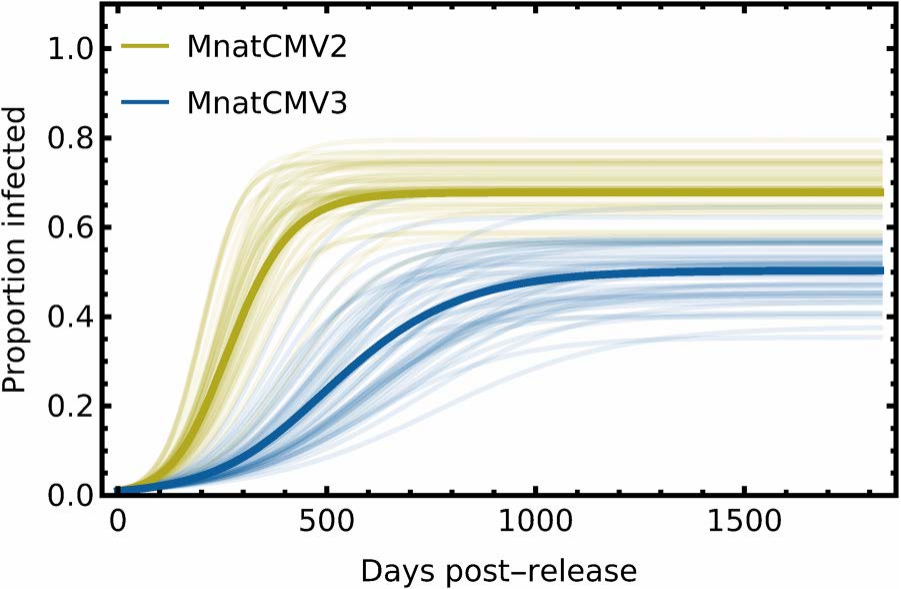
The proportion of animals infected with vaccine over time for simulated releases of vaccines vectored by MnatCMV2 and MnatCMV3. Vaccines were released into completely naive animal populations by direct vaccination of 1% of the animal population. Each faint line indicates the outcome of a single release where parameters were drawn at random from the posterior distribution for the vector virus. The bright lines indicate the mean values across 200 replicate releases for MnatCMV2 and MnatCMV3. Individual outcomes are shown for only 50 simulated releases to maintain visual clarity. Vaccine releases were simulated independently of one another.

Although vector *R*_0_ defines the range of pathogens that can be theoretically controlled by a transmissible vaccine [16], it provides incomplete information on the speed with which immunity to a target pathogen can be established. This is particularly true for virus vectors that alternate between active and latent infection and for which no simple analytical predictions exist. To better quantify the utility of MnatCMV2 and MnatCMV3 as vectors for a transmissible vaccine, we simulated vaccine releases. As expected based on the posterior distributions for *R*_0_, MnatCMV2 reaches, on average, a greater prevalence than MnatCMV3 (Fig 4). On average, MnatCMV2 reaches a prevalence of 0.672 five years after introduction whereas MnatCMV3 reaches a prevalence of 0.505 at this stage. Moreover, simulated vaccine releases demonstrated that MnatCMV2 spreads much more rapidly than MnatCMV3, with MnatCMV2 reaching a prevalence of 0.504 within one year of introduction and MnatCMV3 reaching a prevalence of only 0.124 at the same stage. Thus, although vaccines constructed from both viruses are predicted to ultimately reach prevalences sufficient to eliminate Lassa virus from *M. natalensis* populations, vaccines using MnatCMV2 as a vector should achieve this goal more rapidly than vaccines vectored by MnatCMV3. These results represent the best-case scenario for vaccine spread, conditioned on the assumptions that the entire *M. natalensis* population is fully susceptible to both MnatCMVs at the time of vaccine introduction and that integration of an immunogenic transgene has no impact on vector epidemiology.

### Vector endemicity may necessitate sustained vaccine delivery

**Fig 5:**
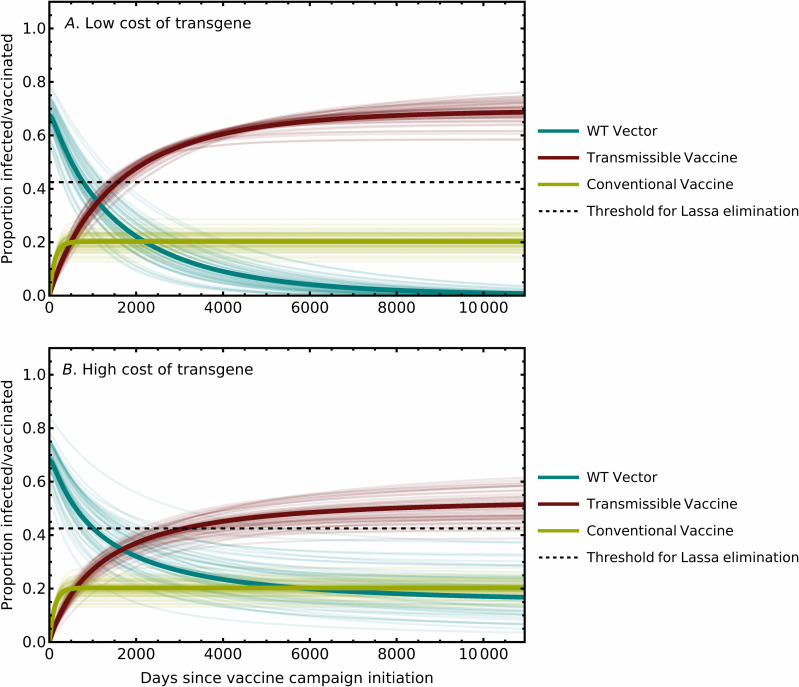
The proportion of animals infected with wild-type vector virus and infected/vaccinated by transmissible vaccine over time compared to the proportion of animals vaccinated by a directly administered conventional vaccine. In Panel A, the transgene is assumed to reduce vaccine transmission by 5% relative to the wild type vector. In Panel B, the transgene reduces vaccine transmission by 15% relative to wild type. Wild type vector virus was allowed to establish and reach a steady state prior to vaccine introduction. Vaccine was then continuously introduced by vaccinating six randomly selected animals per day. Conventional vaccination was simulated independently by administering vaccine to six randomly selected animals per day. Each faint line indicates the outcome of a single simulation where parameters were drawn at random from the posterior distribution. The bright lines indicate the mean values across 200 replicate simulations. Individual outcomes are shown for only 50 simulated releases to maintain visual clarity. The black dashed line indicates the threshold level of vaccination required to eliminate Lassa virus from the *M. natalensis* population based on its estimated upper *R*_0_ in Faranah, Guinea [6].

If the wild-type vector circulates naturally within the reservoir animal population and prevents subsequent infection by the vaccine, repeated introduction is required for a transmissible vaccine to spread [17]. The rate at which introduction must occur grows with the cost of carrying the transgene [17]. To investigate how well a MnatCMV2 vectored vaccine would perform when introduced into a population of *M. natalensis* where MnatCMV2 was endemic and at its steady-state prevalence, we performed simulated releases. These simulated releases assumed that prior infection by MnatCMV2 prevented subsequent vaccine infection and that transgene carriage reduced vaccine transmission relative to the wild type vector. As expected based on previous theory [17], even when repeated vaccine introduction is required, the performance of a transmissible vaccine exceeds that of a conventional vaccine. Specifically, simulations demonstrate that a MnatCMV2 vectored transmissible vaccine experiencing a 5% cost of transgene carriage generally achieves vaccination coverage around three times that of a conventional vaccine when both are administered continuously and at random to reservoir animals at the same rate (Fig 5). Even a transmissible vaccine experiencing a 15% cost of transgene carriage achieves around 2.5 times the vaccine coverage of a conventional vaccine. As the cost of transgene carriage increases, the gains in vaccine coverage conferred by vaccine transmission decrease.

## Discussion

We used sequencing of saliva samples from *M. natalensis* captured in the area of Bafodia, Sierra Leone, to identify animals infected with previously isolated and genetically characterized MnatCMVs and to investigate their potential for use as vectors of transmissible vaccines targeting Lassa virus. Two of the three previously identified MnatCMVs (MnatCMV2 and MnatCMV3) were detected frequently, with prevalences of 72.4 and 49.1%, respectively. Fitting age-structured models of MnatCMV circulation to the infection data using Bayesian MCMC demonstrated that the *R*_0_ of MnatCMV2 (*R*_0_ = 2.99) is greater than that of MnatCMV3 (*R*_0_ = 1.96). This difference in *R*_0_ suggests that a vaccine vectored by MnatCMV2 would yield significantly greater vaccination coverage than one vectored by MnatCMV3, a prediction that was borne out by simulated vaccine releases (Fig 4). The potential utility of MnatCMV2 as a vaccine vector is further supported by our demonstration that co-infection by MnatCMV2 and MnatCMV3 is common and greater than the level predicted under the assumption of neutrality. This finding is consistent with superinfection at the level of different CMVs, a property that facilitates the efficient spread of recombinant vector transmissible vaccines [10,17,18].

Although the estimated *R*_0_ values appear sufficient for the elimination of Lassa virus by vaccines using MnatCMV2 and MnatCMV3 as vectors [6,16], they are smaller than those previously reported for a member of subfamily *Betaherpesvirinae* that circulates within populations of common vampire bats (*Desmodus rotundus*), and which is being pursued as a vector for a transmissible vaccine targeting rabies virus [12,13,19]. Maximum likelihood estimation of viral time series data for a range of mechanistic models led to an estimated *R*_0_ = 6.9 for this virus (*Desmodus rotundus* betaherpesvirus: DrBHV) [19]. Although it is quite possible that DrBHV has a significantly larger *R*_0_ than MnatCMV2 and MnatCMV3, this difference may also be explained by the use of a very small (150 bp) region of the genome to assign distinct viruses rather than the multiple, large regions that we used. Specifically, the greater *R*_0_ value for DrBHV may be attributable to the lumping together of multiple DrBHV-like viruses rather than to the presence of a single virus with a relatively large *R*_0_. This distinction is critical because it is the properties of individual viruses that determine their utility as vectors for recombinant transmissible vaccines.

In addition to quantifying the potential for spread within the *M. natalensis* population, our study clarifies the dynamics of MnatCMV shedding and transmission. By fitting a mathematical model with active (virus shedding at detectable levels) and latent (no detectable virus shedding) states to the age-structured and virus-specific infection data, it was possible to estimate the rate at which animals transition to and from latency. The results indicate that latency is rare, with MnatCMV2 infected animals spending only 7.04% of their time in the latent state and MnatCMV3 infected animals spending 13.66% of their time in this state. This relative rarity of latency in MnatCMV is consistent with results from rhesus cytomegalovirus in rhesus macaques, which tend to exhibit chronic lifelong shedding rather than distinct phases of latency and reactivation [20,21]. Our results are also broadly consistent with data reported for DrBHV in common vampire bat populations from which it was estimated that animals infected with DrBHV spend only 25% of their time in a latent state [19]. Collectively, the data suggest that CMV latency may be infrequent in some wild animals, and that failure to detect infection in saliva samples may be better explained by random variation in saliva quantity and levels of virus shedding.

The results of our analyses suggest that MnatCMV2 is a promising vector for a transmissible vaccine targeting Lassa virus in its natural reservoir, *M. natalensis*. However, this conclusion rests on important assumptions and limitations. First, our study focused on a single population of *M. natalensis* in the area of Bafodia, a small town in northern Sierra Leone. This site was selected based on previous work in adjacent southern Guinea (Faranah) demonstrating that Lassa virus is prevalent within *M. natalensis* and that human infection is extremely common [5,22]. Our focus on deep sampling of a single population comes at the expense of understanding broader geographic variation in virus prevalence, *R*_0_, and the strength of interactions between strains. If the *R*_0_ of MnatCMV2 within *M. natalensis* varies strongly with geography, our results may not generalize well outside the study region. If variation in the *R*_0_ of MnatCMV2 and the *R*_0_ of Lassa virus are driven by shared features of the reservoir animal population, however, they are likely to be positively correlated and our results quite general. Second, we modeled the outcome of vaccine releases under the explicit assumption that the virus used to construct the transmissible vaccine would be initially absent or would not block subsequent infection by the vaccine. If, instead, the virus is already circulating within the target population, interference between vaccine and its parental wild virus may occur, potentially necessitating repeated vaccine introduction [17,18]. Even in this worst case scenario, however, our results demonstrate that a MnatCMV2 vectored transmissible vaccine will still signficantly outperform a conventional vaccine. Third, the model we used to estimate parameters for the vector viruses assumed that the *M. natalensis* population was at steady state and did not show strong seasonal fluctuations in birth or death rates that could have had cascading impacts on viral prevalence. Although this would have been a poor assumption for populations of *M. natalensis* in East Africa [23], our previous work in nearby Faranah, Guinea, revealed only very mild seasonal fluctuation in the reservoir populations [6]. Thus, although weak seasonal fluctuations may occur in Bafodia, they are unlikely to be sufficient to have undermined our steady-state approach. This assumption is further supported by the absence of any detectable difference in the prevalence of MnatCMV2 and MnatCMV3 between wet and dry seasons.

Although our results suggest MnatCMV2 is well-suited to serve as a vector for a transmissible vaccine, engineering the virus to stimulate an immune response against Lassa virus may reduce its ability to spread within reservoir animal populations. This could occur if the addition of an immunogenic transgene reduces viral replication and shedding within the animal or its ability to compete with other MnatCMVs circulating within the reservoir population. Based on prior work, it seems likely that the vaccine will experience at least some cost associated with transgene carriage [24–26]. Because releasing prototype transmissible vaccines is not an option for quantifying these costs, they will need to be estimated using controlled laboratory experiments. Estimates of costs derived in this way can then be integrated into models like those developed here to predict the performance of specific vaccine candidates and to evaluate if they are sufficiently promising for further development and testing.

Looking ahead, we do not yet know whether it will be possible to engineer MnatCMV2 to carry a transgene that yields strong immunity to LASV without compromising transmission. Nonetheless, our estimates of *R*_0_ for MnatCMV2 and MnatCMV3, coupled with the apparent absence of interference between the two viruses, indicate that MnatCMV2 is currently a viable candidate for development as a vector for a transmissible vaccine targeting Lassa virus within its natural host reservoir.

## Methods

### Ethics statement

Rodent sampling was performed according to procedures approved under the University of California, Davis Institutional Animal Care and Use Committee protocol 20849.

### Rodent sampling

Rodents were trapped in and around the town of Bafodia in northern Sierra Leone between July 2019 and February 2021 as described in previous work [27,28]. Briefly, traps were baited and set at dusk inside houses, across town and outside of the town and were checked early in the morning for two consecutive nights per visit. The town was visited a total of five times, with an overall trap effort of 2788 trap-nights and a trapping success of 10%. Captured rodents were transported to a safe location where they were transferred to a ziplock bag by inverting the trap into the bag. A cotton ball with isoflurane or halothane was then placed inside the bag in a tea mesh and the bag sealed. Animals were monitored until fully anesthetized (pedal withdrawal reflex). Once fully anesthetized, the animal was removed from the bag and a blood sample and oral and rectal swab collected. Sex and biometric data were also recorded, along with an initial assignment of species identification made using visual characteristics. Rodents captured within human habitations were euthanized. Rodents captured outside of human habitations were released where they were trapped with the exception of a small subset euthanized for tissue collection. If the animal was going to be euthanized, it was kept in the bag with isoflurane/halothane until a heartbeat was not detected. Rodent sampling was performed according to procedures for BSL3 work in the field [29] under University of California, Davis Institutional Animal Care and Use Committee protocol 20849. All specimens were stored in liquid nitrogen in the field and transferred to a −80 ° C freezer upon arrival at the University of Makeni laboratory. Total nucleic acid was extracted from oral swabs collected in lysis buffer and preserved in liquid nitrogen in the field using MagMAX kits on the KingFisher Duo Prime platform (ThermoFisher Scientific, Waltham, MA, USA). Aliquots were shipped frozen to the University of Glasgow laboratory for sequencing.

Rodents identified as *M. natalensis* using DNA barcoding as part of previous analyses of these animals [27,28] were aged based on measured body-length. Specifically, well-established relationships between eye lens weight (ELW) and age exist for *M. natalensis* [30]. Because ELW data were not collected for the animals in our sample, we instead established a predictive relationship between body-length and ELW using a large, existing data set that included information on body length and ELW for *M. natalensis* animals captured in Guinea [15]. Specifically, we performed a linear regression in R with ELW (mg) as the response variable and body length from snout tip to base of tail (mm) as the single predictor variable (Intercept = -12.21, Slope = 0.284, $p=2.0\times 10^{-16}$, *R*^2^ = 0.75). The resulting fitted model was then used to predict the ELW for animals in our sample and their ages using the established formula. The data file used in all analyses is available on Github (https://github.com/snuismer/Evaluating-native-cytomegaloviruses-as-vectors).

### Virus sequencing

DNA in the total nucleic acid samples was fragmented using an LE220 focused ultrasonicator (Covaris, Woburn, MA, USA) to an approximate size of 450 bp. DNA sequencing libraries were prepared using a Kapa LTP library preparation kit for Illumina platforms (Kapa Biosystems, Wilmington, MA, USA). The library preparation protocol was followed until the adapter ligation stage, at which point the samples were indexed uniquely using NEBNext multiplex oligos for Illumina (96 unique dual index primer pairs) sets 1–4 (New England Biolabs, Ipswich, MA, USA) with either 10 or 16 PCR cycles, dependent on input (10 cycles for samples containing detectable DNA, 16 cycles for samples containing undetectable DNA). The libraries were amplified by following the library preparation protocol, quantified using a Qubit dsDNA HS kit (ThermoFisher Scientific), and analysed using a 4200 Tapestation system (Agilent Technologies, Santa Clara, CA, USA) with high sensitivity D5000 screentape (Agilent) and high sensitivity D5000 reagents (Agilent).

The amplified libraries were sequenced as pools loaded at a final concentration of 1.35 pM using a NextSeq 550 system (Illumina, San Diego, CA, USA) and a NextSeq 500/550 high output v2.5 300 cycle kit (Illumina). Each pool generated approximately 800 million paired end reads, each read consisting of an 8 nt index sequence followed by a 151 nt sequence from the nucleic acid sample. Data from each library sequenced in more than one pool were combined.

### Bioinformatics

Read datasets were trimmed of index reads and quality screened using Trim Galore v.0.4.0 https://www.bioinformatics.babraham.ac.uk/projects/trim_galore/ with parameters –illumina –paired. Trimmed datasets were aligned with a reference consisting of ten individual sequences: a *M. natalensis* mitochondrial genome sequence (GenBank accession MG017593.1; 16,215 nt) to confirm host species identification and three large genome segments each from isolates of MnatCMV1 (OP429138.1; 205,097 nt), MnatCMV2 (OP429139.1; 207,012 nt) and MnatCMV3 (OP429140.1; 211,478 nt) from Mali [11]. These segments contain genes M44-M57, M69-M105 and m128-m160, respectively. All but one of the 15 genes in the M44-M57 segment have orthologues throughout the family *Orthoherpesviridae*. Similarly, 24 of the 37 genes in the M69-M105 segment have orthologues throughout the family. In contrast, none of the 32–37 genes in the m128-m160 segment have orthologues throughout the family, and most orthologues are restricted to genus *Muromegalovirus* (in which the MnatCMVs cluster) in subfamily *Betaherpesvirinae*. The genome locations of the three segments are 42,010–73,557 (31,548 nt), 77,959–129,477 (51,519 nt) and 158,718–197,337 (38,620 nt) in MnatCMV1, 42,965–74,512 (31,548 nt), 78,767–130,336 (51,570 nt) and 160,570–199,328 (38,759 nt) in MnatCMV2 and 39,847–71,182 (31,336 nt), 75,016–127,444 (52,429 nt) and 157,079–201,705 (44,627 nt) in MnatCMV3.

Alignment of trimmed read datasets to the reference was carried out using Bowtie 2 v2.4.2 [31] with the ––no-discordant and ––no-mixed parameters and a maximum fragment length of 1200 nt for valid paired-end alignments. This excluded read pairs for which the reads did not align appropriately with respect to each other in terms of relative orientation and fragment length and read pairs for which only one read aligned. The numbers of reads aligning with each sequence in the reference were recorded by visualising the alignments using Tablet v1.21.02.08 [32]. Reads aligning to each of the three MnatCMV genome segments were standardized by dividing by the number of reads aligning to the *M. natalensis* mtDNA genome sequence. Animals were designated as infected by a specific MnatCMV if they had greater than $1\times 10^{-7}$ standardized reads aligning to the relevant MnatCMV genome for all three MnatCMV segments.

### Model fitting

Key properties of each MnatCMV were quantified by fitting the age-specific infection data to mathematical models describing MnatCMV circulation within an age-structured *M. natalensis* population. Models were fit to each vector virus independently. Animals were assumed to be born at a per capita rate, *b*, with newborn animals entering the susceptible class, *S*, unless produced by an individual in the *I* class that passes the infection vertically with probability σ. Animals experience density-independent mortality at rate μ and density-dependent mortality at rate αn where *n* is the total *M. natalensis* population size. Susceptible individuals that encounter infectious individuals (*I*) become infected at rate β and move into the infectious class *I*. Infectious individuals move into a latent class (*L*) at rate ω in which, although still infected, they no longer shed virus and thus cannot infect other individuals. This model is agnostic about the true nature of the latent class, assuming only that individuals in this class do not actively shed virus. This could be due to true latency (absence of viral replication) or to periods in which viral replication is too low for transmission and detection. Individuals with latent infections spontaneously reactivate and return to the actively infectious class at rate ρ. Assuming the animal population is sufficiently large for stochastic effects to be ignored leads to the following system of partial differential equations describing the distribution of animals of age *a* in each class as a function of time, *t*:


$$\begin{array}{c} \frac{\partial S}{\partial t}=-\frac{\partial S}{\partial a}-\beta SI-\mu S-\alpha Sn \end{array}$$ (1a)

$$\begin{array}{c} \frac{\partial I}{\partial t}=-\frac{\partial I}{\partial a}+\beta SI-\omega I+\rho L-\mu I-\alpha In \end{array}$$ (1b)

$$\begin{array}{c} \frac{\partial L}{\partial t}=-\frac{\partial L}{\partial a}+\omega I-\rho L-\mu L-\alpha Ln \end{array}$$ (1c)

with boundary conditions:


$$\begin{array}{c} S(0,t)=b(\tilde{S}(t)+\tilde{L}(t)+(1-\sigma )\tilde{I}(t)) \end{array}$$ (2a)

$$\begin{array}{c} I(0,t)=b\sigma \tilde{I}(t) \end{array}$$ (2b)

$$\begin{array}{c} L(0,t)=0 \end{array}$$ (2c)

where ˜ indicates the total density of the class across all ages. Although the model assumes parameters are independent of age, including age explicitly significantly increases the accuracy of parameter estimation when age-structured data are available.

MnatCMV sequence data were fit to this model independently for each virus assuming that the system was at steady state. Specifically, the steady state solution to equation (1) was used to solve for the probability that an animal of age *a* was in the actively infectious class, *I* (S1 Text). We focused on the actively infectious class, *I*, because we assume that only this class sheds virus in saliva that is detectable by sequencing of oral swabs. The probability of being in the actively infectious class can then be used in conjunction with the age of each sampled rodent and its infection status inferred from sequence data to calculate the likelihood of the data (S1 Text). This likelihood was integrated into a Bayesian framework, and the posterior distribution was calculated using the Metropolis Hastings algorithm implemented in C++. To facilitate comparison with prior theory demonstrating that vaccine and vector *R*_0_ are important determinants of pathogen control [16,17], we calculated *R*_0_ using a local stability analysis of the virus-absent equilibrium for model 1 and used this value to perform a substitution for the parameter β. Prior distributions for model parameters were selected to be weakly informative and constrained by biological plausibility (S1 Text). Chains were tested for convergence and thinned to remove autocorrelation. Before applying this method to MnatCMVs, it was tested and fine-tuned using simulated data sets. All model parameters, with the exception of vertical transmission σ, were identifiable (S1 Text). Despite the failure to accurately estimate vertical transmission, this parameter was maintained in the model to accurately reflect how this uncertainty influences the estimation of remaining model parameters.

### Interactions between strains

Interactions between MnatCMV2 and MnatCMV3 were investigated by comparing observed levels of co-infection to those predicted by the model, which assumes the viruses are independent. Specifically, simulated data sets were created by drawing parameters at random from the posterior distribution of each MnatCMV and assigning a probability of infection by each virus to each animal using its age and equation (7) of the S1 Text. The probability of infection was then used to assign the animal an infection status (uninfected, infected by MnatCMV2, infected by MnatCMV3, or infected by both MnatCMV2 and MnatCMV3) by drawing at random from a binomial distribution for each virus. For each simulated data set, the average level of coinfection was calculated by summing the number of animals infected by both MnatCMVs and dividing by the total number of animals. This procedure was repeated 2000 times to generate a null distribution for the frequency of co-infection expected if the two viruses do not interact.

### Simulating vaccination of a naive wildlife population

To understand how MnatCMVs are likely to spread, we simulated the introduction of each virus into a completely susceptible population of *M. natalensis*. Parameters were drawn at random from the posterior distribution and the spread of the virus was simulated over a 10 year period after being introduced into 1% of the *M. natalensis* population. Simulations were performed by numerically solving the system of ordinary differential equations:


$$\begin{array}{c} \frac{d\tilde{S}}{dt}=b(\tilde{S}+\tilde{L})+b(1-\sigma )\tilde{I}-\beta \tilde{S}\tilde{I}-\mu \tilde{S}-\alpha \tilde{S}n \end{array}$$ (3a)

$$\begin{array}{c} \frac{d\tilde{I}}{dt}=\sigma b\tilde{I}+\beta \tilde{S}\tilde{I}+\rho \tilde{L}-\omega \tilde{I}-\mu \tilde{I}-\alpha \tilde{I}n \end{array}$$ (3b)

$$\begin{array}{c} \frac{d\tilde{L}}{dt}=\omega \tilde{I}-\rho \tilde{L}-\mu \tilde{L}-\alpha \tilde{L}n \end{array}$$ (3c)

which correspond to the system of partial differential equations (1) when all model parameters are independent of age and ˜ again indicates the total density of the class across all ages. This procedure was repeated 200 times to generate a distribution of outcomes that captures the uncertainty in the estimated parameter values.

### Simulating vaccination of a wildlife population where the vector is endemic

To quantify the benefit of vaccine transmission when the vaccine is introduced into a population of *M. natalensis* where the vector virus is endemic, we simulated a more complex model that allowed for co-circulation of vaccine and vector and continuous vaccine introduction:


$$\begin{array}{c} \frac{d\tilde{S}}{dt}=b(\tilde{S}+{\tilde{L}}_{C}+{\tilde{L}}_{V})+b(1-\sigma )({\tilde{I}}_{C}+{\tilde{I}}_{V})-\beta \tilde{S}({\tilde{I}}_{C}+(1-\kappa ){\tilde{I}}_{V})-\mu \tilde{S}-\alpha n\tilde{S}-\delta \frac{\tilde{S}}{n} \end{array}$$ (4a)

$$\begin{array}{c} \frac{d{\tilde{I}}_{C}}{dt}=b\sigma {\tilde{I}}_{C}+\beta \tilde{S}{\tilde{I}}_{C}+\rho {\tilde{L}}_{C}-\omega {\tilde{I}}_{C}-\mu {\tilde{I}}_{C}-\alpha n{\tilde{I}}_{C} \end{array}$$ (4b)

$$\begin{array}{c} \frac{d{\tilde{L}}_{C}}{dt}=\omega {\tilde{I}}_{C}-\rho {\tilde{L}}_{C}-\mu {\tilde{L}}_{C}-\alpha n{\tilde{L}}_{C} \end{array}$$ (4c)

$$\begin{array}{c} \frac{d{\tilde{I}}_{V}}{dt}=\delta \frac{\tilde{S}}{n}+b\sigma {\tilde{I}}_{V}+(1-\kappa )\beta \tilde{S}{\tilde{I}}_{V}+\rho {\tilde{L}}_{V}-\omega {\tilde{I}}_{V}-\mu {\tilde{I}}_{V}-\alpha n{\tilde{I}}_{V} \end{array}$$ (4d)

$$\begin{array}{c} \frac{d{\tilde{L}}_{V}}{dt}=\omega {\tilde{I}}_{V}-\rho {\tilde{L}}_{V}-\mu {\tilde{L}}_{V}-\alpha n{\tilde{L}}_{V} \end{array}$$ (4e)

where the subscripts C and V indicate wild type vector virus and vaccine virus, respectively, and the ˜ again indicates the total density of the class across all ages. The parameter κ quantifies the reduction in vaccine transmission, relative to the wild type vector virus, attributable to carriage of the immunogenic transgene and the parameter δ quantifies the rate at which animals are directly inoculated with transmissible vaccine. This model was simulated without vaccine for ten years, allowing the vector virus to reach its endemic steady-state. After the initial ten-year period, vaccine introduction began at rate δ and continued throughout the simulated vaccination campaign. Vector virus and reservoir animal parameters were drawn at random from the posterior distribution for each combination of vaccine cost (κ) and vaccine introduction rate (δ). This procedure was repeated 200 times for each combination of κ and δ to generate a distribution of outcomes reflecting the uncertainty of model parameter estimates.

## Supporting information

S1 Text Supporting derivations, analyses, and figures. This supporting file includes derivations of the likelihood function, simulation testing of the estimation procedure, and posterior distributions for model parameters.
(PDF)
