# Supplementary material for: Evaluating vectors for the design of a spillover-disrupting Lassa virus transmissible vaccine
Source: PLoS Comput Biol. 2026 Jun 26;22(6):e1014390. doi: 10.1371/journal.pcbi.1014390 (PMC13322505; doi:10.1371/journal.pcbi.1014390)
Supplement: S1 Text — This supporting file includes derivations of the likelihood function, simulation testing of the estimation procedure, and posterior distributions for model parameters. (PDF) [file pcbi.1014390.s001.pdf]

Supporting online material: Evaluating vectors for the design of a spillover-disrupting Lassa virus transmissible vaccine

Age estimation

Because eye lenses were not collected as part of our field study in Bafodia, we used data previously collected from nearby *M. natalensis* populations in Guinea [1] to establish a relationship between animal body length and eye lens weight (ELW). Specifically, we performed a linear regression in R with ELW (mg) as the response variable and body length from snout tip to base of tail (mm) as the single predictor yielding a linear model with intercept equal to -12.21 and slope equal to 0.284. Both intercept and slope were highly significant ($p < 2.0 \times 10^{-16}$) and the model explained considerable variation in the data ($R^2 = 0.75$). This relationship was then used to predict ELW from body length. Finally, *M. natalensis* age was calculated by applying the well-established formula:

$$a = exp\left[\frac{10.46088 + ELW/2}{4.35076}\right] \quad (1)$$

where a is the predicted age in days and ELW is the weight of a pair of eye lenses in mm [2].

Derivation of the likelihood function

Steady-state force of infection

Assuming all rates are age-independent, model (1) in the main text can be reduced to the ODE system

$$\frac{dS}{dt} = b(S + L + (1-\sigma)I) - \beta SI - \mu S - \alpha S\hat{n} \tag{2a}$$
$$\frac{dI}{dt} = b\sigma I + \beta SI + \rho L - \omega I - \mu I - \alpha I\hat{n} \tag{2b}$$
$$\frac{dL}{dt} = \omega I - \rho L - \mu L - \alpha L\hat{n} \tag{2c}$$

where $\hat{n} = \frac{b-\mu}{\alpha}$. Solving for the steady state of this system yields the following prediction for the abundance of individuals in the actively infectious class:

$$\hat{I} = \frac{(b+\rho)\big(b(\beta - \alpha(1-\sigma)) - \beta\mu\big) - b\alpha\omega}{\alpha\beta(b+\rho+\omega)}. \tag{3}$$

Thus, at steady state, the force of infection is given by:

$$\hat{\lambda} = \beta\hat{I} = \frac{b(R_0 - 1)((b+\rho)(1-\sigma) + \omega)}{b+\rho+\omega} \tag{4}$$

where the expression has been re-written in terms of the composite parameter $R_0 = \dfrac{\beta\hat{n}(b+\rho)}{b((b+\rho)(1-\sigma)+\omega)}$ to simplify interpretation of fitted models.

Probability of being actively infected as a function of age

The solution for the steady-state force of infection can be used to predict the probability that an individual of age a is in the I class and thus, by assumption, actively shedding virus detectable by sequencing of oral swabs. Specifically, at steady state, the probability of an individual of age a being in each class (S, I, and L) conditioned on being alive is given by the following system of ordinary differential equations:

$$\frac{dS}{da} = -\lambda S \tag{5a}$$
$$\frac{dI}{da} = \lambda S + \rho L - \omega I \tag{5b}$$
$$\frac{dL}{da} = \omega I - \rho L \tag{5c}$$

where the initial conditions are:

$$S(0) = 1 - \sigma \frac{\hat{I}}{\hat{n}} \tag{6a}$$
$$I(0) = \sigma \frac{\hat{I}}{\hat{n}} \tag{6b}$$
$$L(0) = 0 \tag{6c}$$

Solving this system for $I(a)$ yields the probability that an individual is in the actively infectious class as a function of age:

$$\mathcal{P}(a) = \frac{\exp[-\lambda a]\big(\exp[\lambda a]\rho(\rho + \omega - \lambda) + K_1 - K_2\big)}{(\rho + \omega)(\rho + \omega - \lambda)} \tag{7}$$

where:

$$K_1 = \frac{(b + \lambda)(\lambda - \rho)(\rho + \omega)((\sigma - 1)(b + \rho) - \omega)}{(b + \rho)(b(\sigma - 1) - \lambda) - \omega(b + \lambda)} \tag{8a}$$
$$K_2 = \frac{\lambda\omega \exp[a(\lambda - \rho - \omega)](b + \rho + \omega)(-\sigma(b + \rho) + b + \lambda)}{(b + \rho)(b(-\sigma) + b + \lambda) + \omega(b + \lambda)} \tag{8b}$$

are constants introduced only to allow expression (7) to be easily displayed on a single line and λ is given by the steady-state force of infection equation (4).

The likelihood function

Solution (7) can then be used to calculate the likelihood of observing a sample of individuals with specific ages and infection statuses:

$$\mathcal{L} = \sum_{i=1}^{N} \mathcal{P}(a_i)^{x_i} + (1 - \mathcal{P}(a_i))^{1-x_i} \tag{9}$$

where x_i is the infection status of animal i inferred from sequencing and a_i is the age of animal i and the summation is taken over the N *M. natalensis* animals in the sample.

Parameter estimation

Model parameters were estimated using Bayesian MCMC. Specifically, we implemented the Metropolis Hastings algorithm in C++ using the likelihood function (9) and a multivariate normal proposal distribution. Prior distributions for model parameters were selected using a combination of available data and biological plausibility (Table 1). Five chains were run for 1,000,000 steps with a burn in of 300,000 steps. Chains were thinned to retain one value for every 50 steps to reduce temporal autocorrelation. Convergence was assessed by comparing variance within and among chains (e.g., Gelman Rubin criterion), visual comparison of posterior distributions across chains, and quantification of temporal autocorrelation within chains. The C++ source code is available in the GitHub repository (https://github.com/snuismer/Evaluating-native-cytomegaloviruses-as-vectors).

Table 1: Prior distributions for model parameters

Parameter	Interpretation	Prior
b	Per capita birth rate	Lognormal with mode $\frac{1}{a}$ and standard deviation 0.2
R_0	Basic reproductive number	Lognormal with mode $\frac{1}{1-\text{prevalence}}$ and standard deviation 1.0
ω	Rate of latency	Exponential with rate 20
ρ	Rate of reactivation	Exponential with rate 20
σ	Probability of vertical transmission	Beta with $\alpha = 1.0$ and $\beta = 10.0$

Testing and fine tuning using simulated data

Before applying our Bayesian inference method to our data set, we tested and refined the method using simulated data sets. Specifically, we simulated 100 data sets using the steady state solution (7) with parameters drawn at random from the distributions defined in Table 2 and animal ages drawn from an exponential distribution with mean $1/b$. To test our method more rigorously, animal ages were censored so that only animals 21 days and older could be sampled as is the case in the real data where *M. natalensis* younger than this age are not yet weaned and thus impossible to trap [3]. The sample size in simulated data sets was

Table 2: Parameter distributions used for data simulation.

Parameter	Interpretation	Distribution
b	Per capita birth rate	U$[\frac{1}{700},\frac{1}{200}]$
R_0	Basic reproductive number	U[2,10]
ω	Rate of latency	U$[0,\frac{1}{21}]$
ρ	Rate of reactivation	U$[0,\frac{1}{21}]$
σ	Probability of vertical transmission	U$[0,\frac{1}{5}]$

identical to that in the real data set.

Each simulated data set was analyzed using our Bayesian method and the estimated parameter values (modal value of univariate posterior) regressed against the true parameter values used to generate the simulated data. Repeated iterations of data simulation and method testing were performed to optimize proposal distributions. Ultimately, we selected a multivariate normal proposal distribution with off-diagonal elements (covariances) set to zero and diagonal elements (variances) given by the following values: $V_b = 1 \times 10^{-6}$, $V_{R_0} = 5.0$, $V_\omega = 1 \times 10^{-3}$, $V_\rho = 1 \times 10^{-3}$, $V_\sigma = 0.01$. After refining proposal distributions through this iterative process, we evaluated the fit between estimated parameter values and their true values to quantify identifiability. Reliability of 95% highest posterior density (HPD) credible intervals was assessed for each parameter by calculating the percentage of cases in which the true parameter value was within the predicted HPD. Below, we discuss our ability to estimate each model parameter in turn.

Estimation of R_0

Simulation testing demonstrates that R_0 can be estimated with reasonable accuracy as long as the true value does not become too large (Figure 1). The reason R_0 becomes increasingly challenging to estimate as its true value increases is that large values of R_0 cause most individuals to become infected at a young age. Thus there exists little discernible difference in the age structure of infected individuals for large values of R_0, particularly when individuals cannot be sampled prior to weaning as we have assumed here. Nevertheless, the 95% HPD reported by our method includes 99% of true values, demonstrating that the credible intervals produced by our method for R_0 are reliable across a broad range of parameters.

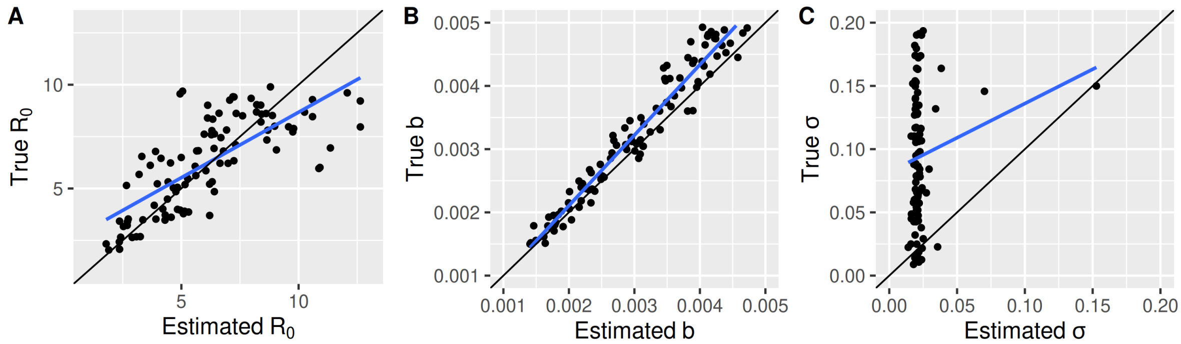

Figure 1: The true value of the parameters R_0 (A), b (B), and σ (C) used to simulate data (y-axis) versus their estimated values (x-axis). The blue line is the least squares fit whereas the black line is the 1:1 line demarcating a perfect fit between true and estimated values. For R_0, the intercept of the best fit line was 2.70, the slope was 0.57, and $R^2 = 0.54$. For b, the intercept of the best fit line was 0.00, the slope was 1.12, and $R^2 = 0.95$. For σ, the intercept of the best fit line was 0.08, the slope was 0.54, and $R^2 = 0.02$. The inability of our method to estimate σ, the probability of vertical transmission, is unsurprising given the constraint that animals cannot be sampled until they are older than 21 days.

Estimation of b

The birth rate of the rodent population is estimated accurately (Figure 1) and the true value lies within the 95% HPD in 100% of cases.

Estimation of σ

Based on our comparison of true and estimated values, the probability of vertical transmission is not identifiable (Figure 1). The challenges associated with estimating σ are unsurprising given the nature of the real and simulated data sets where animals could not be sampled until they were weaned and had emerged from the nest. Thus, the clearest signal of vertical transmission – infection very early in life – was invisible to our approach. Nevertheless, the true value of σ fell within the 95% credible interval predicted by our method in 100% of cases. We included the parameter σ, despite our inability to estimate it accurately, to ensure that we explicitly accounted for uncertainty in the degree to which vertical transmission of MnatCMV occurs.

Table 3: Parameter estimates and credible intervals for MnatCMV2 (columns 2-3)and Mnat CMV3 (columns 4-5).

Parameter	Estimate	Credible interval	Estimate	Credible interval
b	0.007	0.004-0.01	0.007	0.005-0.01
R_0	2.99	2.22-4.27	1.96	1.58-2.65
ω	0.002	2.32×10^{-5}-0.023	0.004	8.75×10^{-7}-0.047
ρ	0.059	3.80×10^{-4}-0.238	0.070	1.42×10^{-3}-0.226
σ	0.015	6.45×10^{-7}-0.221	0.012	2.18×10^{-6}-0.181

Estimation of ω and ρ

The rate at which active infections become latent, ω, and the rate at which latent infections reactivate, ρ, are estimated with reasonable accuracy (Figure 2). Both quantities, however, are consistently overestimated. Importantly, however, as with R_0, the true values of ω and ρ are reliably within their predicted 95% credible interval. Specifically, the true values of ω and ρ are within their 95% HPD in 100% of cases. Thus, although considerable noise exists in point estimation, credible intervals are reliable across a broad range of parameter values.

Although estimates for the parameters ω and ρ are noisy, these quantities themselves are of less interest than the composite parameters, $P_A = \frac{\rho}{\rho+\omega}$ and $P_L = \frac{\omega}{\rho+\omega}$ which quantify the probability that an infected animal is actively infected and shedding virus or latently infected without virus shedding, respectively, conditioned on being alive. Comparing the true and estimated values of these quantities reveals that both can be estimated with good accuracy (Figure 2).

Application to real data

We applied our Bayesian method to the MnatCMV data set, analyzing each virus independently. Because MnatCMV1 was not detected in animals sampled in and around Bafodia, Sierra Leone, we applied the method to only MnatCMV2 and MnatCMV3. Posterior distributions for model parameters are show in Figure 3. Modal values and 95% HPD credible intervals were calculated in R and are shown in Table 3.

References

[1] Fichet-Calvet, E. *et al.* Diversity and dynamics in a community of small mammals in coastal Guinea, West Africa. *Belgian Journal of Zoology* **139**, 93–102 (2009). WOS:000276349200002.

[2] Marien, J. *et al.* Evaluation of rodent control to fight Lassa fever based on field data and mathematical modelling. *Emerging Microbes & Infections* **8**, 640–649 (2019).

[3] Nuismer, S. L. *et al.* Bayesian estimation of Lassa virus epidemiological parameters: Implications for spillover prevention using wildlife vaccination. *Plos Neglected Tropical Diseases* **14**, e0007920 (2020). WOS:000574557800007.

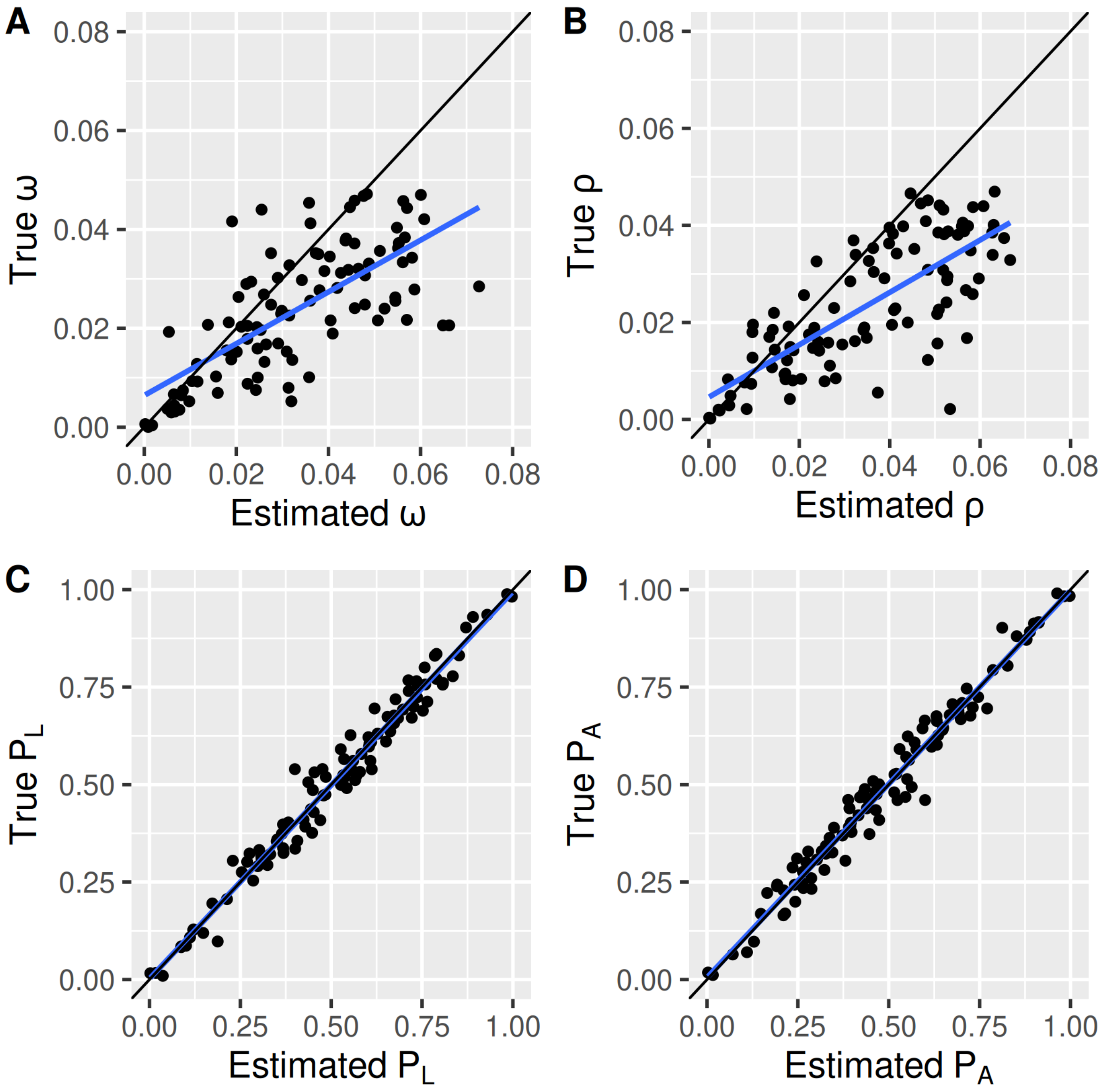

Figure 2: The top row shows the true value of the parameters ρ (panel A) and ω (panel B) used to simulate data (y-axis) vs. their estimated values (x-axis). The bottom row shows the true (x-axis) and estimated (y-axis) values of the composite parameters $P_A = \frac{\omega}{\omega+\rho}$ (panel C) and $P_L = \frac{\rho}{\omega+\rho}$ (panel D) which quantify the proportion of time an infected individual spends in the actively infectious state or the latent state, respectively. In all panels the blue line is the least squares fit whereas the black line is the 1:1 line demarcating an idealized perfect fit between true and estimated values. For ω, the intercept of the best fit line was 0.01, the slope was 0.52, and $R^2 = 0.50$. For ρ, the intercept of the best fit line was 0.00, the slope was 0.54, and $R^2 = 0.57$. For P_L, the intercept of the best fit line was 0.00, the slope was 0.99, and $R^2 = 0.97$. For P_A, the intercept of the best fit line was 0.00, the slope was 0.99, and $R^2 = 0.97$.

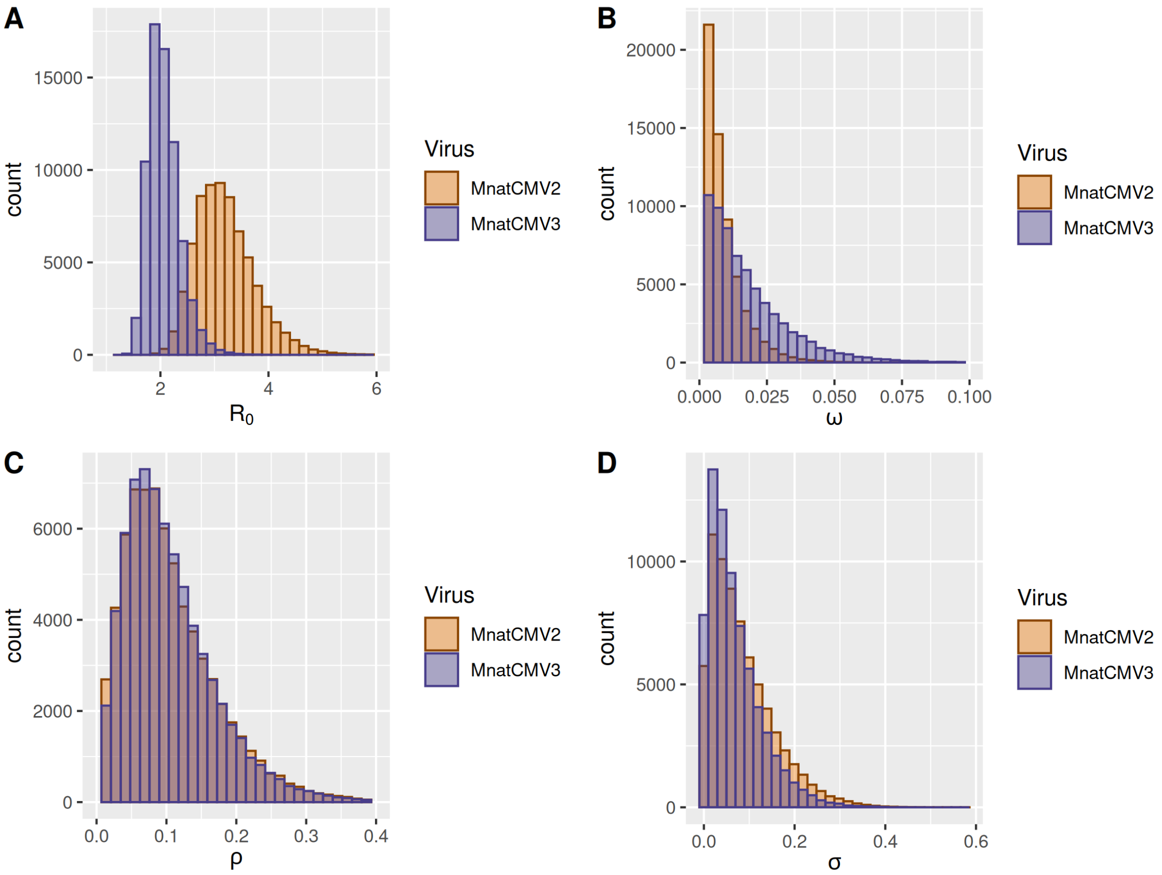

Figure 3: Posterior distributions for model parameters R_0 (A), ω (B), ρ (C), and σ (D). Posterior distributions were estimated using Bayesian MCMC applied to age-structured *M. natalensis* infection data.
